# A Highly Selective GSK-3β Inhibitor CHIR99021 Promotes Osteogenesis by Activating Canonical and Autophagy-Mediated Wnt Signaling

**DOI:** 10.3389/fendo.2022.926622

**Published:** 2022-07-18

**Authors:** Bo Wang, Saima Khan, Pengtao Wang, Xiaofang Wang, Yangxi Liu, Jingjing Chen, Xiaolin Tu

**Affiliations:** Laboratory of Skeletal Development and Regeneration, Institute of Life Sciences, Chongqing Medical University, Chongqing, China

**Keywords:** CHIR99021, Wnt/β-catenin signaling, autophagy, integrated 3D printing, PCL scaffold, osteoporosis, slow-release hydrogel, osteoblast differentiation

## Abstract

The discovery and application of small molecules is one of the practical strategies of safe osteogenic drugs. The small molecule CHIR99021 (C91) is a highly specific, safe, and most effective GSK-3β Inhibitor. This study found that it efficiently activates the canonical Wnt signaling of bone marrow stromal cell ST2 and promotes osteoblast differentiation and mineralization. C91 increases the production and biochemical activity of osteoblast marker alkaline phosphatase, the expression of osteoblast marker genes *Alpl, Bglap, Runx2*, and *Sp7*, and the formation of bone nodules. Triptonide is a transcription inhibitor of Wnt target gene, which diminishes C91-induced osteoblast differentiation in a dose-dependent manner. Meanwhile, C91 also induces autophagy through autophagosome formation and conversion of autophagy biomarker LC-3I into LC-3II. Autophagy inhibitor 3MA partially reduces C91-induced osteoblast differentiation and mineralization; autophagy inducer Rapamycin increases the expression of β-catenin to promote osteogenic differentiation, but cannot alleviate the inhibition of Triptonide on C91-induced osteogenic differentiation, indicating the crosstalk of canonical Wnt signaling and autophagy regulates C91-induced osteoblast differentiation. Furthermore, in order to simulate the *in vivo* detection of C91 in osteogenesis process, we made a C91 slow-release hydrogel with our newly established polycaprolactone and cell-integrated 3D printing system (PCCI3D module). The sustained release C91 promotes the differentiation and mineralization of ST2 cells. C91 can also enhance the proliferative activity of ST2 cells. The release rate of C91 from hydrogel gradually decreases within 7 days. During this period, the C91 is released by 83.0% and the cell viability maintained at 96.4%. Therefore, the small molecule Wnt agonist C91 promotes osteogenesis through caonical and autophagy-mediated Wnt signaling pathway with an option for translational application.

## 1 Introduction

Clinically, osteogenic drugs teriparatide (PTH), abaloparatide (PTHrP) and romosozumab (sclerostin antibody) (1, 2) are all proteins with side effects, narrow treatment window, and small population of patients. Therefore, it is still necessary to continue to find safe osteogenic drugs. PTH, PTHrP, and romosozumab are all proteins with high price, easy degradation, short half-life, immunogenicity, supraphysiological dosage for patients, and the large molecular weight, which affects their biological activity, reduces their availability, and produces tissue permeability (3). These adverse factors limit their use to a certain extent. Small molecules can minimize these limitations of macromolecules such as proteins. For example, small molecules act usually more stable, and soluble, easier to process, less denatured, and more suitable for commercialization because of their low cost (3). In addition, the action of small molecules is fast and reversible. The diversity of structure and function endows small molecules with more applicable potential (4). Therefore, small molecule drugs that stably stimulate bone formation are gradually becoming the research hotspot and development goal of new drugs for the treatment of osteoporosis.

Wnt signaling is the top of the five developmental signaling pathways, which is popular and most fruitful (5). Wnt signaling can be divided into canonical and non-canonical pathways, which control the cell fate in embryonic stage and postnatal life, including cell proliferation, differentiation, polarization, and migration (6). β-catenin is the central player of canonical Wnt signaling and activated by the inactivation of GSK-3β kinase upon Wnt binds its receptor Frizzled and co-receptor low-density lipoprotein (Lrp) 5/6. This leads to the accumulation of free non-phosphorylated β-catenin in the cytoplasm and its translocation into the nucleus, where the β-catenin cooperating with the transcription factor Tcf/Lef activates Wnt target gene expression. Therefore, the activity of the Wnt/β-catenin signaling pathway is dependent on the amount of β-catenin in the cytoplasm and GSK-3 plays a key role in controlling the amount of β-catenin.

Multiple GSK-3α/B kinase inhibitors were shown to increase bone mass. Lithium is a GSK-3β kinase inhibitor, and increases bone mineral density in a study of 26 patients treated with LiCl for more than 10 years (7). Treatment of oral gavage with LiCl on accelerated aging and osteoporotic SAMP6 mice and their control C57BL/6 mice displays significantly improved bone mass in both strains of mice (8). Lithium has been widely used for many years in the treatment of bipolar disorder and other psychiatric disorders, but its effects on calcium homeostasis are complex. Lithium therapy produces goiter and hypothyroidism, and may also lead to hyperparathyroidism (7, 9). Especially, long-term use of lithium will continue to increase PTH, elevates bone resorption, resulting in bone loss. It may also affect calcium transport at the tubular and intestinal levels as well (10). In addition, due to the low toxicity of chloride, long-term use will also have harmful effects on bones.

It is necessary to search for new agents of GSK-3β inhibitors for clinical use. Another FDA-approved GSK-3β inhibitor Tideglusib, commonly used in the clinical treatment of Alzheimer’s disease, resulted in a significant increase in new bone formation of fractured mice along with enhanced cortical bone bridging and medullary bone deposition. However, the drug produces an increase in serum aminotransferase levels (11). In addition, many inhibitors are not selective for GSK-3 and also inhibit cyclin-dependent protein kinases (CDKs) because of their homologous similarities of 33% amino acid sequence with GSK-3.

CHIR99021 (C91) is a highly selective GSK-3 inhibitor, does not inhibit CDKs (12). Most of the reports about GSK-3 inhibitors are ATP competitive inhibitors, and this class of inhibitors act on the ATP binding site of GSK-3 with generally good *in vitro* activity. But this ATP binding site is highly conserved among more than 500 kinases, and the selectivity of ATP competitive inhibitors is poor. So, C91 is by far the most potent and specific GSK-3 inhibitor against GSK-3α、β subunits with miraculous effects (13). However, whether C91 can promote osteoblast differentiation and its underpinning mechanisms are the focus of this study.

In ZDF rats, a single oral dose of C91 rapidly lowered plasma glucose (14). C91 rescued Gaucher disease iPSC in osteoblast differentiation and bone matrix deposition through Wnt signaling (15). But there has been no report about C91 in osteogenesis. This study tests the hypothesis that C91 promotes osteogenic differentiation of bone marrow stromal cell line ST2, and rapidly validate it’s *in vivo* osteogenic function by our newly-established technology of integrated 3D printing of hard materials and cells, as well as with a drug sustained-release system.Recently, autophagy showed an involvement in osteogenic differentiation and bone formation induced by Wnt signaling (16). Autophagy is an important way of maintaining eukaryotic cell balance by breaking down and discarding damaged proteins and organelles (17, 18). The lack of autophagic function in osteoblasts reduces mineralization capacity and induces an imbalance of osteoblast and osteoclast populations, resulting in a low bone mass phenotype (17). The relationship between Wnt signaling and autophagy is through regulation of β-catenin activity to regulate autophagic activity (18). But how the crosstalk between C91-activated Wnt signaling and autophagy works needs further investigation.

In this study, we found that the small molecule C91 promotes osteogenic differentiation of bone marrow stromal cells *via* the activation of Wnt signaling, which also activates autophagy. In addition, activation of autophagy alone can upregulate the expression of β-catenin to promote osteogenic differentiation. However, autophagy cannot reverse the inhibition of Wnt signaling antagonist Triptonide on C91-induced osteogenic differentiation (**Figure 8F**). To evaluate *in vivo* function of C91, a 3D printed artificial bone scaffolds by using our newly established integrated 3D printing of polycaprolactone and cells (19) verified that slow releasing C91 on 3D modules promotes the osteogenic differentiation and mineralization of ST2 cells with good survival rate and growth of ST2 cells. Thus, the small molecule Wnt agonist C91 has translational application value in maintaining bone marrow stromal cell for osteogenesis through canonical and autophagy-mediated Wnt signaling.

## 2 Materials and Methods

### 2.1 Reagents and Cells

#### 2.1.1 Chemicals

Wnt signaling agonist CHIR99021 (C91), Wnt signaling inhibitor Triptonide (Tript), and autophagy inhibitor 3MA were purchased from MedChemExpress (Shanghai, China). Lyophilized gelatin methacryloyl (GelMA) and photo-cross-linking agent (LAP) from Sunp biotech (Beijing, China), polycaprolactone (PCL) from Sigma (St. Louis, MO, USA), Trizol from Invitrogen (Thermo Fisher Scientific, Carlsbad, CA, USA), and Alizarin red S staining Kit from Solarbao Biotechnology (Beijing, China).

#### 2.1.2 Assay Kits

BCIP/NBT alkaline phosphatase (AP) color-rendering kit, live/dead viability assay kit, alkaline phosphatase activity assay kit, DAPI staining solution, and monodansycadaverine (MDC) staining kit were obtained from Beyotime Biotechnology (Shanghai, China), AG RNAex Pro Reagent AG21101, SYBR^®^ Green Pro Taq HS Master Mixed qRT-PCR Kit II AG 11702, and reverse transcription reaction kits AG11705 from Accurate Biotechnology (Hunan, China), and CCK-8 assay kit from MedChemExpress (Shanghai, China).

#### 2.1.3 Antibodies

polyclonal anti-β-catenin antibody, GAPDH antibodies and polyclonal anti-LC-3I/II antibody were supplied by Wanlei (Shenyang, China), and FITC labeled goat anti-rabbit IgG (H+L) from Beyotime Biotechnology (Shanghai, China).

#### 2.1.4 Reagents of Cell Culture and Cell Line

Penicillin, α-MEM, and streptomycin were purchased from Gibco (Beijing, China), fetal bovine serum (FBS) from Biological Industries (Israel), Wnt3a-expressing cells and control L cells from the American Type Culture Collection (ATCC) (Manassas, VA, USA). ST2 cells were kindly obtained from Dr. Steve Teitelbaum, Washington University.

### 2.2 Cell Culture

The cell culture of murine cell line ST2, Wnt3a-expressing cells, and control L cells was performed as previously reported (20). These cells were cultured in 90% α-MEM,10% FBS supplemented with 50 U/mL penicillin, and 50 μg/mL streptomycin. DMSO and C91-treated ST2 cells were cultured in each well of a 24-well plate at a density of 4x10^4^ cells.

### 2.3 Activation and Inhibition of Wnt Signaling in ST2 Cells

C91 was used to trigger the Wnt signal pathway of ST2 cells. In the meantime, ST2 cells treated by DMSO was used as the control. Triptonide was utilized to obstruct the Wnt signaling pathway *via* its binding to the C-terminal transactivation domain of β-catenin to stop β-catenin activity (21). To stimulate Wnt signaling in ST2 cells, ST2 cells was splited in each well of a 24-well plate at a density of 4x10^4^ cells and treated with C91 at concentrations of 5μM for 72 hours or as indicated time. As for measuring the specificity of the role of Wnt signaling in ST2 cells on osteoblast differentiation, ST2 cells was treated with C91 at 5 μM, followed by Triptonide treatment (10nM) for 72 hr until the end of the experiment.

### 2.4 PCL, C91, and Cell-Integrated 3D Printing (PCCI3D)

3D printing was performed as reported (19), GelMA hydrogel was used to laden cells and C91 drags.10 μM of C91 and 2 x 10^5^ ST2 cells were each mixed with 0.25 mL of α-MEM. Cells and C91 were individually mixed with an equal volume of GelMA solution for 3D bioprinting. The cell-loaded GelMA solution and the C91-loaded GelMA solusion were reciprocally printed out at 25°C as cell beams in a diameter of 300μm with an interval of 500μm between cell beams at a printing speed of 5 mm/s (19). After the printing of one layer, the GelMA hydrogels were crosslinked under blue light at 405 nm for 10 s. Then, printing was continued for another layer. After three layers of printing, a PCL、C91 and cell-integrated 3D (PCCI3D) module was fabricated.

### 2.5 Cell Viability Assay

Cell viability in PCCI3D modules was quantitated by a live/dead viability assay kit. After 1, 4, and 7 days of cell culture as reported (22), the cells were washed with PBS and incubated in the staining mixture, prepared by adding 1 μL Calcein AM (1000X) and 1 μL PI (1000X) in 1 mL assay buffer. Calcein AM was used to dye the living cells, and PI was used to stain the dead cells. The plates were incubated in the incubator for 0.5 hr, and the samples were imaged immediately using an inverted fluorescence microscope (LEICA, Germany). Cell viability was quantified using the ImageJ software (64-bit v1.46).

### 2.6 Cell Proliferative Activity

As reported (22), the Cell Counting Kit-8 kit (CCK-8, Beyotime, China) was used to evaluate the proliferation activity of cells in the functional modules at day 1, 4, and 7 step by step. For this assay, modules were washed with PBS once, and divided into 4 pieces, and placed in a 96-well plate. Next, 90 μL PBS and 10 μL CCK-8 solution were added to each well followed by 2 hr incubation. Subsequently, the supernatant was pipetted into a new 96-well plate, and a microplate reader’s absorbance at 450 nm was measured.

### 2.7 RNA Extraction and Gene Expression Analysis

The total RNA extraction and quantitative PCR (qRT-PCR) were performed. Briefly, total RNA was extracted from the cells with Trizol as previously described (23). The cDNA was synthesized by using high capacity cDNA reverse transcription kit (Aikerui, China) and used as a template for qRT-PCR with primer sets (**Table 1**). Relative mRNA expression levels were normalized to the housekeeping gene glyceraldehyde-3-phosphate dehydrogenase (GAPDH) by using the 2 ^−△CT^ method.

**Table 1:** Sequences of primers used for qRT-PCR (mouse).

Primer	Forward	Reverse
*GAPDH*	GGAGCGAGATCCCTCCAAAAT	GGCTGTTGTCATACTTCTCATGG
*Alpl*	ACACCAATGTAGCCAAGAATGTCA	GATTCGGGCAGCGGTTACT
*Runx2*	TGGTTACTGTCATGGCGGGTA	TCTCAGATCGTTGAACCTTGCTA
*Sp7*	CCCTTCTCAAGCACCATTGG	AAGGGTGGGTAGTCATTTGCATA
*Bglap*	CACTCCTCGCCCTATTGGC	CCCTCCTGCTTGGACACAAAG
*Lef1*	CCTACAGCGACGAGCACTTTT	CCTTGCTTGGAGTTGACATCTG
*Axin2*	TGAGCGGCAGAGCAAGTCCAA	GGCAGACTCCAATGGGTAGCT
*lc3b*	AGAGCGATACAAGGGGGAGA	TTCGGAGATGGGAGTGGACA

### 2.8 Alkaline Phosphatase Staining

The Alkaline Phosphatase Staining (AP staining) was performed as previously described (20). After 3 days of cell culture, the cells were washed with PBS (Sorlabio, Beijing, China) and fixed in 3.7% formaldehyde (Chuandong, Chongqing, China) for 5 min at room temperature. AP staining was performed on the cells for 30 min by using the BCIP/NBT alkaline phosphatase color development kit. It should be noted the ST2 cells in the PCCI3D module are stained after 7 and 14 days, and the staining time is extended to four hours. A digital camera recorded the staining results.

### 2.9 AP Biochemical Activity Assay

The AP biochemical activity assay was performed as previously described (20). After 0.3 mL 10 mM Tris/HCl (pH7.4) was added to each well, the cells were removed from the plate by scratching. The whole process was done on ice. After 3 min centrifugation at 12,000 rpm, the supernatant was used for assay with the AP detection kit step by step per the described procedures (Beyotime, China).

### 2.10 Detection of Translocation of β-catenin Into the Nucleus

ST2 cells were cultured on the 24-well plate treated with C91 or DMSO. After 48 hours, they were washed with PBS, fixed with 4% paraformaldehyde, permeabilized for 30 min with PBS containing 0.25% Triton X-100 (PBS-T), and blocked with 1% BSA in PBS. Immunostaining was performed using the rabbit polyclonal anti-mouse β-catenin antibody (1:500), and FITC labeled goat anti-rabbit IgG (H+L) secondary antibody (1:500) as described. Samples were washed three times in PBS, the last one of which included a 5 min incubation in DAPI (1:5000), and washed again (24). Then images were acquired using a fluorescence microscope.

### 2.11 Matrix Mineralization Assay (Alizarin Red S Staining)

As previously reported that Alizarin Red S Staining (ARS; Cyagen Biosciences) was performed to assess late mineralization (20). ST2 cells were seeded in 6-well plates and cultured with C91. After 14 days of culture, cells were fixed in 4% formaldehyde for 10 min at room temperature. Finally, the cells were treated with ARS (2%, pH 7.5) for 30 min and then rinsed with distilled water. PCCI3D modules were firstly cultured in the growth medium for seven days. The mineral nodule formation was induced in the osteogenic medium containing 0.1 mM dexamethasone, 10 mM β-glycerophosphate disodium salt, and 50μg/mL L-ascorbic acid another 14 days. The modules were stained with alizarin red S for 30 min and photographed under a microscope.

### 2.12 Western Blot Analysis

As previously reported (25). Cells were lysed in RIPA buffer supplemented with protease and phosphatase inhibitors (Beyotime). Equal amounts of proteins were separated by 10% SDS-PAGE and then transferred onto polyvinylidene flfluoride (PVDF) membranes (Millipore, Shanghai, China). After blocking in 5% non-fat milk for 2 h, the membranes were incubated overnight at 4°C with antibodies specific to GAPDH (1:1,500; CST#5174, Cell Signaling Technology, Danvers, MA, United States), β-catenin (1:1,000; ab32572, Abcam), LC-3I/II(1:1,000; ab128025, Abcam). After washing four times (5 min each time) in Tris-buffered saline with 0.1% Tween 20 (TBST), the membranes were incubated with horseradish peroxidase-conjugated secondary anti-mouse or anti-rabbit antibodies (Beyotime) for 1 hr at room temperature. After washing three times (5 min each time) with TBST, proteins were detected using enhanced chemiluminescence blotting reagents (Millipore). Signal intensity was measured using a Bio-Rad XRS chemiluminescence detection system (Bio-Rad, Hercules, CA, United).

### 2.13 Monodansycadaverine (MDC) Staining

As previously reported that MDC staining was commonly used for the detection of autophagosomes (25). The cells were grown in 12-well plates, then treated with C91 and Earle’s Balanced Salt Solution (EBSS reagent, an autophagy activator). After 48 h, they were stained with 50 μM of MDC, a selective fluorescent marker for autophagic vacuoles, at 37°C for 30 min. The cellular fluorescence changes were observed using a fluorescence microscope (EU5888; Leica, Wetzlar, Germany).

### 2.14 C91 Release Experiment in PCCI3D Module

As previously reported (26). C91 solutions were prepared at concentrations of 0.1 mg/mL, 0.2 mg/mL, 0.4 mg/mL, 0.6 mg/mL, 0.8 mg/mL, 1.0 mg/mL, 1.2 mg/mL and 1.4 mg/mL, and then the absorbance values were measured at a wavelength of 276 nm in a UV spectrophotometer (C91 drug solution has a maximum absorbance peak at a wavelength of 276 nm). The absorbance peak of C91 solution at wavelength 276 nm) and the standard curve of C91 concentration-absorbance value were made. The PCCI3D Module was immersed in 2 mL of pure water and incubated in an incubator at 37°C. At each time point, 1 mL of supernatant containing C91 was collected, 1 mL of fresh purified water was added back to the vial, and then the absorbance value of the supernatant containing C91 was detected at a wavelength of 276 nm. Finally, the amount of C91 released from the PCCI3D Module per day was calculated from the standard curve.

### 2.15 TopFlash/FopFlash Fluorophore Reporter Gene Analysis

As previously reported (27). ST2 cells were cultured into 24-well plates, and the Wnt/β-catenin signaling reporter TopFlash and FopFlash plasmids (Addgene, Cambridge, MA, USA) were cotransfected into ST2 cells using Lipofectamine 3000 (Invitrogen). The transfected ST2 cells were then treated with C91, L-ctrl, and Wnt3a conditioned media. At 72 hr post-transfection, luciferase analysis was performed using a luciferase reporter analysis system (Promega) according to the manufacturer’s instructions. Results are expressed as normalized TopFlash/FopFlash values, and results are expressed as the mean ± standard deviation (SD) of three replicates.

### 2.16 Statistical Analysis

Statistical analysis was performed by GraphPad Prism 8.0.1 software. Each experiment was repeated three times independently. The data was presented as mean ± SD. The normal distribution of dates was confirmed using Normality and Lognormality Tests. One-Way ANOVA was used to analyze the difference between multiple groups. Meanwhile, Brown-Forsythe and Welch ANOVA tests are used for correction analysis. A T-test was performed among two comparable groups. A nonparametric test was used when the above statistical methods didn’t work. *P <*0.05 was considered a significant difference.

## 3 Results

### 3.1 Effect of C91 on Osteogenic Differentiation of ST2 Cells

**Figure 1:**
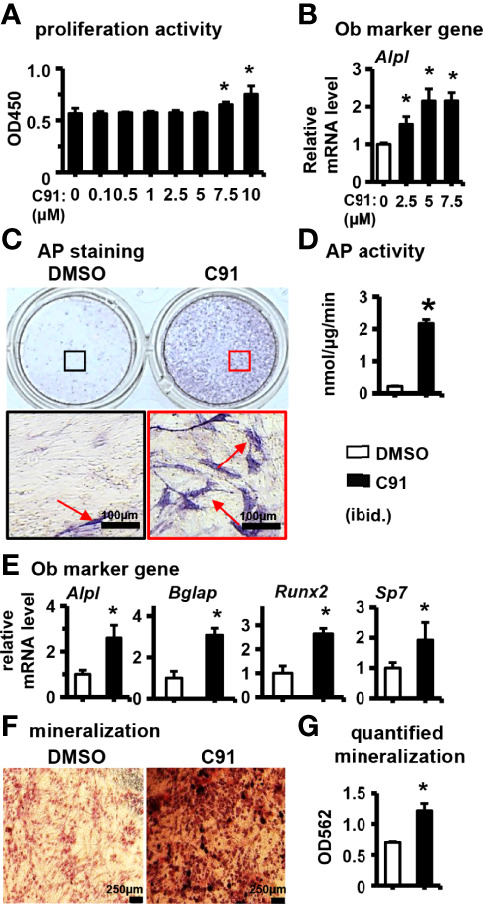
The effect of C91 on ST2 cell differentiation and mineralization. **(A)** The effect of C91 on the proliferation activity of ST2 cells was detected by CCK8 assay. Results are expressed as relative OD450 values, calculated by normalizing OD450 readings to the mean value of the control group. C91 at concentrations of 0.1–5 μM had no significant effect on the viability of ST2 cells. **(B)** qRT-PCR detection of mRNA expression of early osteogenesis marker gene *Alpl*. ST2 cells were treated with C91 (5 μM) for 3 days, followed by AP staining **(C)** analysis and AP biochemical quantification **(D)** analysis. Red arrows indicate alkaline phosphatase staining positive cells. Scale bar = 100 μm. **(E)** The mRNA expression levels of osteogenic target genes were detected by qRT-PCR. **(F)** ST2 cells were treated with C91 for 14 days, and the osteogenic induction medium was changed every two days in between. Alizarin Red S staining was performed (original magnification, x4). Red arrows indicate mineralized nodules, scale bar = 250 μm. **(G)** is the quantitative analysis result of panel **(D)** Results are presented as mean ± SD (n = 3 per group). * indicates *P* < 0.05, compared with the DMSO group.

The effect of different concentrations of C91 on the proliferative activity of ST2 cells was first determined. C91 at concentrations below 5 μM was found to have no significant effect on the proliferative activity of ST2 cells within 72 hr, whereas C91 at 7.5 and 10 μM elevated the cell proliferative activity (**Figure 1A**). C91 at 2.5, 5, and 7.5 μM significantly increased the expression of osteoblast marker gene *Alpl*, compared with the control group. C91 at 2.5 and 5 μM dose-dependently increased osteoblast differentiation, and C91 at 7.5 μM was not continuing to increase *Alpl* gene expression (**Figure 1B**). Therefore, 5 μM was selected as the working concentration of C91 for this study.

After treatment of ST2 cells with 5 μM C91 for 72 hr, AP staining visualized many purple cells as positive differentiation, while a few cells were stained positive in the control group (**Figure 1C**). The AP enzyme activity was robustly increased in the C91-treated group than in the control (**Figure 1D**). C91 at 5 μM significantly upregulated the expression of osteoblast marker genes *Alpl, Bglap, Runx2*, and *Sp7* in ST2 cells (**Figure 1E**). Bone nodule formation is an important marker of mineralization of type I collagen produced by osteoblasts. The osteoinductive function of C91 on ST2 cells was assessed by Alizarin Red S staining. The mineralized nodules produced in ST2 cells were significantly increased in the C91 group compared with the control group (**Figure 1F**), and after quantification, it was increased by 73.0% (**Figure 1G**). The above results indicate that C91 induces the differentiation of ST2 cells into osteoblasts.

### 3.2 Effect of C91 on Wnt/β-catenin Signaling Pathway in ST2 Cells

**Figure 2:**
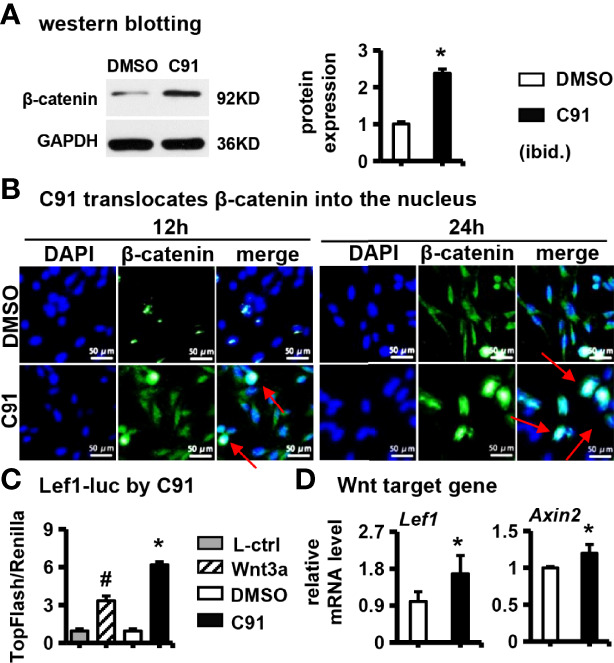
Effects of C91 on Wnt/β-catenin signaling pathway in ST2 cells. **(A)** ST2 cells were treated with C91 for 12 and 24 hours, and the samples were collected for immunofluorescence staining analysis of β-catenin. The red arrows indicate the protein of β-catenin into the nucleus. Scale bar = 50 μm. **(B)** Western blot detection of protein β-catenin expression. **(C)** TopFlash is a reporter gene plasmid used to detect the level of β-catenin-mediated TCF/LEF transcriptional activity in Wnt signaling pathway, and FopFlash plasmid is its negative control. ST2 cells were transfected with TopFlash and FopFlash plasmids and then treated with C91, L-ctrl and Wnt3a conditioned medium for 3 days. Samples were collected and assayed for luciferase activity. **(D)** qRT-PCR detection of Wnt target gene expression. Results are expressed as mean ± SD (n = 3 per group). * indicates *P* < 0.05, compared with the DMSO group. ^#^ indicates *P* < 0.05, compared with the C91 group.

Next we interrogated the effect of C91 on the Wnt/β-catenin pathway in ST2 cells. C91 (5 μM) stabilized the expression of β-catenin protein compared with the control (**Figure 2A**). DAPI fluorescently labeled nuclei blue and anti-β-catenin antibody fluorescently labeled β-catenin green in the cells. The fluorescence intensity of β-catenin protein in the C91-treated group was significantly stronger than those in the control group at 12 hr and 24 hr after C91 stimulation, β-catenin protein was expressed in the cytoplasm and nucleus, and GFP-labeled β-catenin merged with blue fluorescently labeled nucleus, showing blue-green fluorescence (**Figure 2B**). We further determined the effect of C91 on the transduction of the canonical Wnt signaling pathway. Lef1-luciferase reporter assay showed that, as expected, C91 significantly upregulated TopFlash luciferase activity by 548.0% compared with the control group, and the fluorescent plum activity of the C91 group was also elevated by 85.0% than Wnt3a positive control group (**Figure 2C**). qRT-PCR assay showed that C91 enhanced the expression of Wnt target gene *Lef1* and *Axin2* expression, respectively (**Figure 2D**). In summary, C91 activates the canonical Wnt/β-catenin signaling pathway in ST2 cells.

### 3.3 Effect of Inhibiting Wnt/β-catenin Signaling Pathway on Osteogenic Differentiation of ST2 Cells Induced by C91

**Figure 3:**
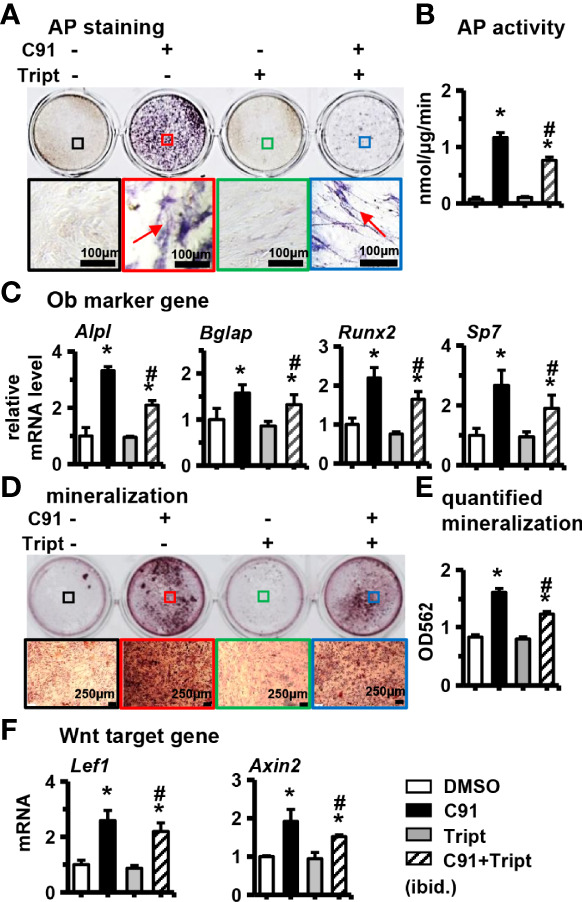
Effects of inhibition of Wnt/β-catenin signaling pathway on ST2 cell differentiation. Triptonide (Tript) is a Wnt signaling inhibitor. ST2 cells were treated with C91 and Tript (10nM) for 3 days, and the samples were collected for AP staining analysis **(A)** and AP biochemical quantitative analysis **(B)**. Positive cells, scale bar = 100 μm. **(C, F)** qRT-PCR detection of osteogenic target genes and Wnt target gene mRNA expression levels. **(D)** ST2 cells were treated with Tript (10 nM) and C91 for 14 days, and the osteogenic induction medium was changed every two days in between. Alizarin red S staining was used to detect mineralized nodules. The lower image is a 10× enlargement of the upper image. Red arrows are mineralized nodules, scale bar = 50 μm. **(E)** is the quantitative analysis result of panel **(D)** Results are expressed as mean ± SD (n = 3 per group). * indicates *P* < 0.05, compared with the DMSO group. ^#^ indicates *P*<0.05, compared with the C91 group.

Triptonide (Tript) is a specific blocker of the Wnt/β-catenin signaling pathway, and Triptonide blocks the Wnt/β-catenin signaling pathway through the C-terminal transactivation domain of β-catenin (28). ST2 cells were pretreated with 10 nM of Triptonide for 1 hr in advance, and then C91 was added to induce differentiation of the cells for 3 days, and then detected AP biochemical activity. The results showed a decrease in the detection of AP staining of the C91-stimulated ST2 cells treated with Triptonide as compared to the C91 treatment alone, and a 34.7% decrease in AP enzyme activity (**Figures 3A, B**). Compared with the C91 group, the expression of osteoblast marker genes (*Alpl, Bglap, Runx2*, and *Sp7*) and Wnt target genes (*Lef1* and *Axin2*) were all decreased at C91 and Triptonide conjunction treatment (**Figures 3C, F**). Alizarin red S staining results of the 14-day culture demonstrated that the C91 group had more brownish-black mineralized nodules than control group. Meanwhile, the number of mineralized nodules induced by C91 and Triptonide conjunction treatment was significantly decreased by 46.0% than that in C91 group (**Figures 3D, E**). These results suggest that Wnt/β-catenin pathway plays a specific and important role in regulating C91-induced osteogenic differentiation.

### 3.4 Effect of C91 on Autophagy in ST2 Cells

**Figure 4:**
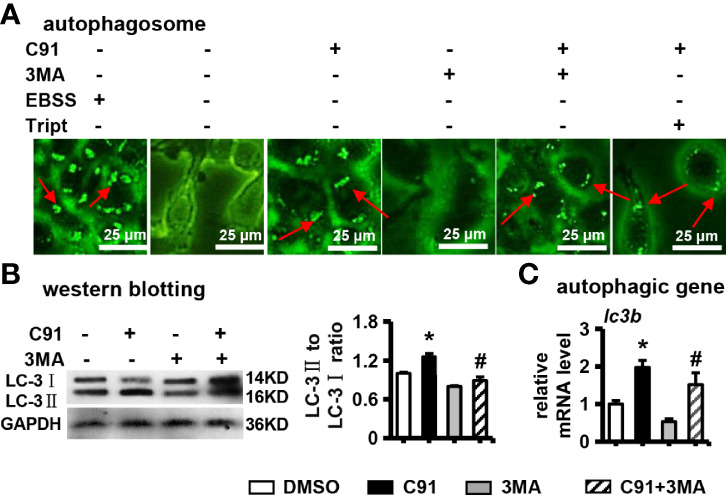
The effect of C91 on autophagy in ST2 cells. **(A)** 3MA is an autophagy inhibitor. ST2 cells were pre-treated with 3MA (5 mM) for 6 hours, and then induced by C91 for 3 days. MDC staining was performed and autophagy was analyzed by fluorescence microscopy. Body formation. Red arrows indicate autophagosomes, scale bar = 25 μm. **(B)** Western blot detection of the expression of autophagy-related protein LC-3I/II. GAPDH was used as an internal control, and the histogram on the right is the quantification of the band on the left. **(C)** qRT-PCR analysis of the mRNA expression of autophagy-related gene *lc3b*. Results are expressed as mean ± SD (n = 3 per group). * indicates *P* < 0.05, compared with the DMSO group. ^#^ indicates *P* < 0.05, compared with the C91 group.

Recently, it has been shown that autophagy is involved in osteogenic differentiation and bone formation induced by Wnt signaling (29). Next, whether autophagy is activated during C91-induced osteogenic differentiation of ST2 cells was investigated. 3MA has been shown to inhibit the progression of autophagy by preventing the formation of autophagosomes through the inhibition of type III phosphoinositide 3-kinase (PI3K) (30). Autophagy is typically characterized by increased formation of AVOs, which represents lysosomes and autolysosomes. Autophagosomes appeared as green punctate by MDC staining. As shown in **Figure 4A**, the formation of autophagosomes labeled with MDC staining was observed at C91-treated ST2 cells, but not in the control group, and 3MA partially inhibited the formation of autophagosomes activated by C91. These results indicated that C91 activated the autophagic response in the treatment of ST2 cells. One marker of autophagic activity in cells, that is, isoforms of LC-3, LC-3I/II. After induction of autophagy, LC-3I becomes acylated (it is converted to LC-3II) and inserts into the autophagic body membrane (31). Compared with the control group, the LC-3II/LC-3I ratio was significantly increased in the C91 group, which represents the most critical event in autophagosome formation. However, the LC-3II/LC-3I ratio was significantly reduced in the C91 and 3MA treatment group (**Figure 4B**). As expected, the expression of autophagy-related factor *lc3b* in the C91 group was higher than that in the control group (**Figure 4C**), and it is worth noting that 3MA inhibits the upregulated expression of *lc3b* by C91. The above results indicate that C91 activates autophagy in ST2 cells.

### 3.5 Effect of Inhibiting Autophagy on Osteogenic Differentiation of ST2 Cells

**Figure 5:**
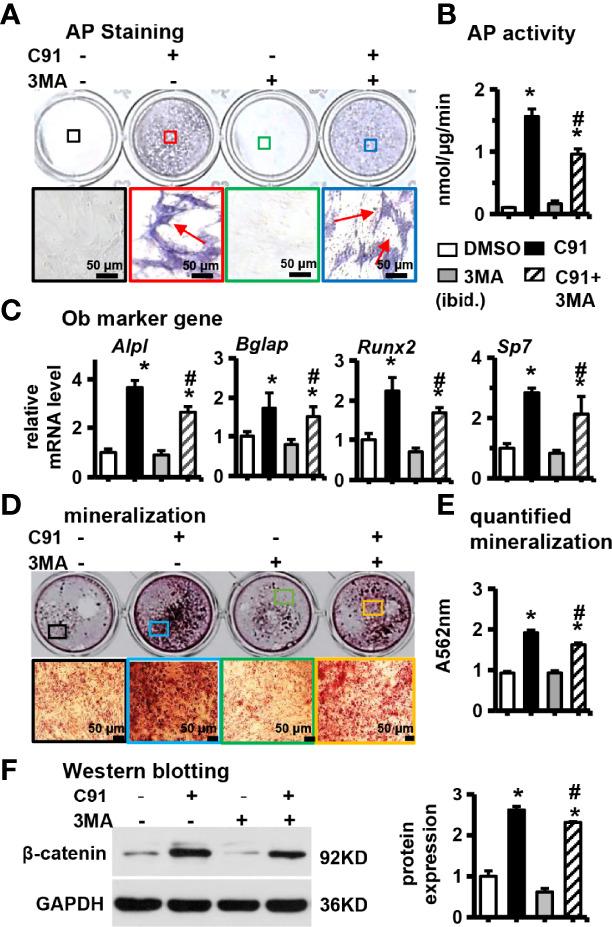
Effects of inhibition of autophagy on ST2 cell differentiation. 3MA is an inhibitor of autophagy. Before ST2 cells were treated with C91, they were pre-treated with autophagy inhibitor 3MA (5 mM) for 6 hours. The samples were collected for AP staining **(A)** analysis and AP biochemical quantitative **(B)** analysis. The bottom panel is 10× magnification of the upper panel, red arrows are alkaline phosphatase-positive cells, scale bar = 50 μm. **(C)** qRT-PCR analysis results. **(D)** ST2 cells were pre-treated with autophagy inhibitor 3MA for 6 hours, and then ST2 cells were treated with C91 for 14 days, and the osteogenic induction medium was changed every two days. Alizarin red S staining was used to detect mineralized nodules. The lower image is a 10× enlargement of the upper image. Scale bar = 50 μm. **(E)** is the quantitative analysis result of panel **(D, F)** The expression of protein β-catenin was detected by Western blot, and the bar on the right is the quantitative result of the band on the left. Results are expressed as mean ± SD (n = 3 per group). *P<0.05, compared to the DMSO group. ^#^ *P*<0.05, compared to the C91 group.

ST2 cells were pretreated with 3MA at a concentration of 5 mM for 6 hr, and then C91 was added to induce differentiation for 72 hr. The results showed that there were more AP-positive cells in the C91 group, while the number of AP-positive cells decreased in the C91 plus 3MA group (**Figure 5A**). Compared with the C91 group, AP biochemical activity of the C91 plus 3MA group was reduced by 65.0% (**Figure 5B**). In addition, the mRNA expression of the C91 plus 3MA group osteogenic differentiation marker genes (*Alpl, Bglap, Runx2*, and *Sp7*) was lower than that of the C91 group (**Figure 5C**). There were many brownish-black mineralized nodules in the C91 group, while the number of mineralized nodules in the C91 and 3MA group was decreased by 30.2% (**Figures 5D, E**). Reducing autophagy also decreased the protein content by 11.7% of β-catenin induced by C91 (**Figure 5F**). In summary, C91 induces autophagy, but autophagy only takes a partial role in the C91-induced osteoblast differentiation.

### 3.6 Relationship Between Autophagy and Wnt/β-catenin Signaling Pathway

Wang et al. reported that C2S NPs induce autophagy by activating mTOR/ULK1, which further induces Wnt/β-catenin signaling pathway to promote bone formation and osteoblast differentiation in BMSCs (32). Consistent with these results, autophagy inhibitor 3MA partially inhibited the expression of β-catenin induced by C91 in ST2 cells (**Figure 5F**). We next tested whether specifically induced autophagy can remove the inhibition of Triptonide on C91-induced osteoblast differentiation.

**Figure 6:**
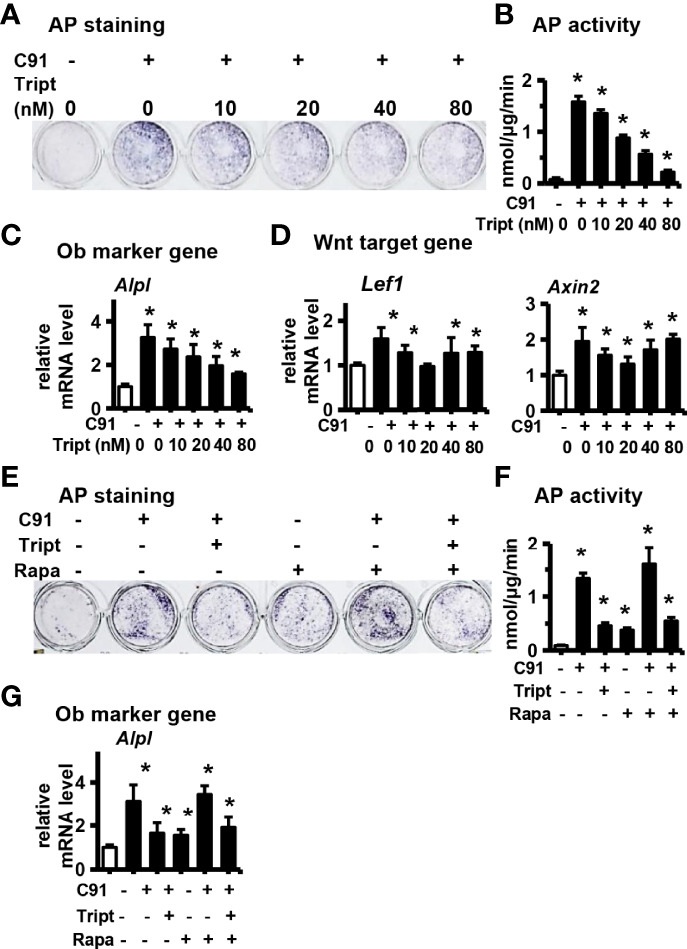
Study the relationship between autophagy and Wnt/β-catenin signaling pathway. ST2 cells were treated with Wnt signaling inhibitors Triptonide(Tript) (10nM, 20nM, 40nM, 80nM) and C91 (5μM) for 3 days, and the samples were collected for AP staining analysis **(A)** and AP biochemical quantitative analysis **(B)**. **(C, D)** qRT-PCR detection of osteogenic target gene and Wnt target gene mRNA expression. Tript at a concentration of 80nM inhibited the osteogenic differentiation of ST2 the best. Rapamycin (Rapa) is an autophagy activator. ST2 cells were treated with C91, Tript (80nM) and Rapamycin (200nM) for 3 days, and the samples were collected for AP staining analysis **(E)** and AP biochemical quantitative analysis **(F)**. **(G)** The expression of Alpl mRNA, an early osteogenic factor, was detected by qRT-PCR. Results are expressed as mean ± SD (n = 3 per group). * indicates *P*<0.05, compared with DMSO group.

The effects of different concentrations of the Triptonide (Tript), a transcription inhibitor of Wnt target gene, on C91-induced osteogenic differentiation of ST2 cells was determined. Triptonide inhibited C91-enhanced AP biochemical activity in a dose-dependent manner (**Figures 6A, B**). Triptonide at a concentration of 80 nM reduced C91-induced AP activity by 90.2%. Similarly, Triptonide decreased the expression of *Alpl*, an early osteogenic factor induced by C91, in a dose-dependent manner (**Figure 6C**). Triptonide at concentrations of 10, 40 and 80 nM decreased the expression of Wnt target gene *Lef1* compared to C91-treated group. Meanwhile, Triptonide at 10 and 20 nM decreased the expression of *Axin2* induced by C91 (**Figure 6D**).

Rapamycin can activate autophagy by inhibiting the mTOR signaling pathway (33, 34). Rapamycin treatment alone did not improve the osteogenic differentiation function of Triptonide in inhibiting C91-induced ST2 cells, as shown by the number of AP-positive cells, AP biochemical activity as well as its gene expression (**Figures 6E, F, G**). Taking together, the autophagy activator Rapamycin did not rescue the reduction of osteogenic differentiation induced by C91 by the Wnt target gene transcription inhibitor Triptonide. So although increasing autophagy alone can increase osteogenic differentiation to a certain extent, inhibiting Wnt signaling also inhibits autophagy -induced osteogenic differentiation function because autophagy promotes osteogenic differentiation by increasing Wnt/β-catenin signaling (**Figure 8F**).

### 3.7 Effect of C91 on the Growth and Proliferation of ST2 Cells in PCCI3D Module

**Figure 7:**
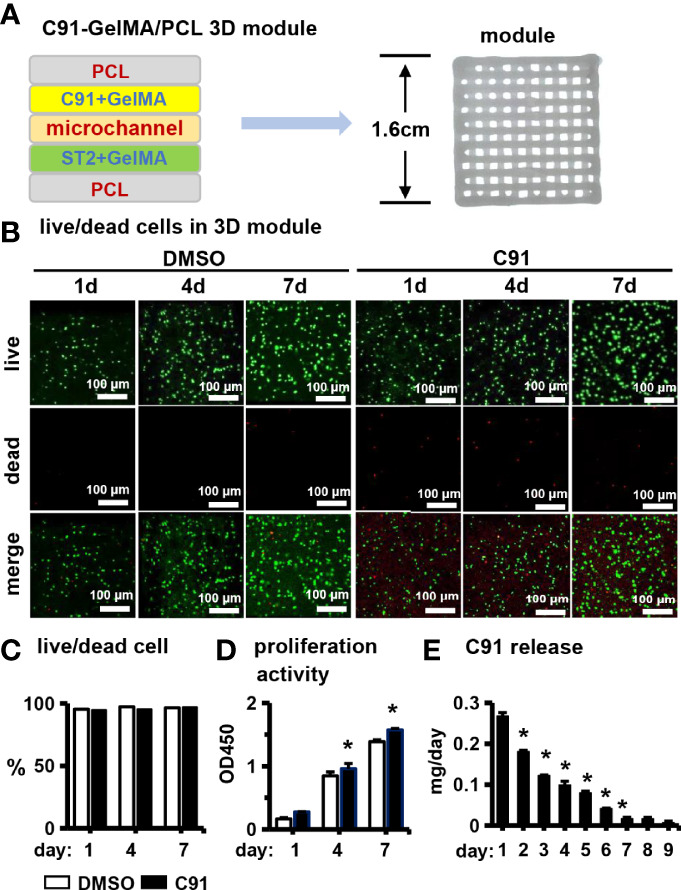
Effects of C91 on ST2 cell growth and proliferation in 3D modules. **(A)** Schematic diagram of a PCI3D system design (left) and its printing module image (right). The C91-loaded hydrogel bundles, the ST2-loaded hydrogel bundles, and the PCL bundles were printed with each other to form one layer, and 4 layers were continuously printed to form a 3D module. **(B)** Live/dead cell staining was used to detect the survival and proliferation of cells 1, 4, and 7 days after *in vitro* culture. Live cells show calcein-am positive (green) and dead cells show EthD-1 negativity (red). Scale bar = 100 μm. **(C)** Live/dead cell ratios were analyzed using confocal microscopy imaging. **(D)** The effect of C91 in the CCK8 detection module on the proliferation activity of ST2 cells. Results are expressed as relative OD450 values, calculated by normalizing OD450 readings to the mean value of the control group. **(E)** The daily sustained release of C91 in the 3D module was detected using a drug release assay. * indicates *P* < 0.05, compared with the DMSO group.

Finally, we validated *in vivo* osteogenesis function of C91, the PCCI3D module was printed with our newly established PCL and cell-integrated 3D printing (PCI3D) system (19) to simulate the real *in vivo* osteogenesis process. The PCL bundle is printed at the bottom of the module, the GelMA hydrogel bundle loaded with ST2 cells is printed in the middle, and the GelMA hydrogel bundle loaded with C91 is printed in the upper part, and the three bundles are printed alternately for a total of 4 layers. The PCCI3D module has a stable support and tunnel connection structure, and the pore size is appropriate, which can be used for the transport of nutritional and metabolic materials (**Figure 7A**).

We first examined the biological activity of the PCCI3D module. The PCCI3D modules were cultured for 1, 4, and 7 days, followed by Calcein AM and PI staining for live/dead cell experiments. ST2 cells in both experimental and control groups exhibited high cell viability at 96.4% (**Figures 7B, C**). The results of CCK8 proliferation activity assay showed that the proliferation ability of control and C91-treated ST2 cells increased linearly (**Figure 7D**). The sustained release C91 promotes the differentiation and mineralization of ST2 cells. C91 can also enhance the proliferative activity of ST2 cells. The release rate of C91 from hydrogel gradually decreases within 7 days (**Figure 7E**). During this period, the C91 is released by 83.0% and the cell viability maintained at 96.4%. Therefore, the small molecule Wnt agonist C91 promotes osteogenesis through caonical and autophagy-mediated Wnt signaling pathway with an option for translational application.

### 3.8 Effect of C91 on Differentiation and Mineralization of ST2 Cells in PCCI3D Module

**Figure 8:**
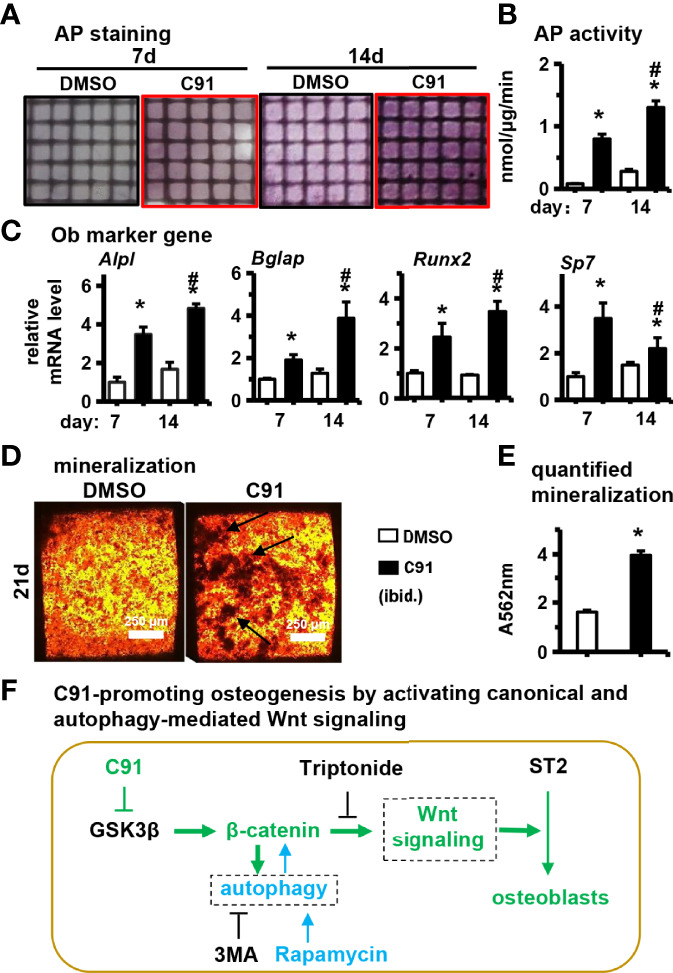
Effects of C91 on ST2 cell differentiation and mineralization in 3D modules. 3D modules were cultured in complete medium for 2 days and then switched to osteogenic induction medium. The medium was changed in half every two days. After 7 and 14 days of culture, AP staining **(A)** analysis and AP biochemical quantification **(B)** analysis were performed. **(C)** qRT-PCR detection of the effect of C91 in the 3D module on the expression of osteogenic target genes in ST2 cells. **(D)** 3D modules were induced to mineralize for 21 days, followed by Alizarin Red S staining to detect mineralized nodules. Black arrows indicate mineralized nodules. Scale bar = 50 μm. **(E)** is the quantitative analysis result of panel **(D, F)** C91-promoting osteogenesis by activating canonical and autophagy-mediated Wnt signaling. *Indicates *P*<0.05, compared to the 7-day DMSO group. # indicates *P*<0.05, compared with the 14-day DMSO group.

We further evaluated the effect of C91 on ST2 cell differentiation in PCCI3D module. The PCCI3D modules were osteogenically induced for 7 and 14 days, respectively, followed by AP staining and biochemical activity assay. Compared with the control group, the differentiation of ST2 cells was significantly increased in the C91 group in PCCI3D Module (**Figures 8A, B**). This was further confirmed by qRT-PCR, where gene expression of osteoblast marker genes such as *Alpl, Bglap, Runx2*, and *Sp7* was significantly increased in the C91 group compared to the control group (**Figure 8C**). To assess the effect of C91 on bone nodule formation in PCCI3D module, The PCCI3D Modules were cultured in osteogenic induction medium for 14 days. Alizarin red S staining showed that the C91 group in PCCI3D module promoted the formation of mineralized nodules in ST2 cells compared with the control group (**Figure 8D**). After quantification, it increased by 145.3% (**Figure 8E**). Taking the above data together, we confirmed the promoting effect of C91 on osteogenic differentiation and mineralization of ST2 cells in PCCI3D Module.

## 4 Discussion

The discovery and application of small molecule is one of the strategies to develop safe osteogenic drugs. This study found that the small molecule GSK-3β inhibitor C91 stabilizes β-catenin, which is translocated in the nucleus of bone marrow stromal cell line ST2 to activate canonical Wnt/β-catenin signaling pathway for osteogenic differentiation and mineralization. Its functional specificity may be evidenced by inhibiting Wnt target gene transcription. Meanwhile, C91 also induces autophagy, and the activation of autophagy alone still needs the upregulation of β-catenin expression to promote osteogenic differentiation. These results suggest that C91-activated autophagy is involved in osteoblast differentiation induced by canonical Wnt signaling. Finally, we reconstructed a function module of PCL and ST2 cells with C91 slowly-released 3D hydrogel (PCCI3D) by our newly developed hard materials and cell integrated 3D printing technology, simulating osteogenesis *in vivo* and validating the osteogenic effect of C91. As osteoblasts are the only cell type of bone formation, understanding the role and mechanism of small molecules in regulating osteoblast differentiation and activity will enrich the knowledge of bone physiology and be conducive to the transformation and application of small molecules.

Although C91 inhibits GSK-3 activity and activates canonical Wnt signaling, reports that C91 directly promotes osteoblast development and differentiation have not emerged. GSK-3 is a protein kinase that phosphorylates a range of substrates and plays a key role in a variety of human diseases. Examples include glycogen synthase (type II diabetes), NFkB/CBP transcription (inflammation), AP-1/NFAT (cardiac hypertrophy), and Tau/APP (neurological disorders) as well as Wnt and Notch signaling (bone homeostasis and vascular calcification and cancer). After clarifying the role of GSK-3 in insulin signaling, several inhibitors that are effective, specific, and do not inhibit CDKs have been developed, such as C91 (14, 35) and AR-A014418 (36). They activate glycogen synthase, deposit glycogen in the liver, rapidly reduce glucose and insulin concentrations, and lower fasting blood glucose levels. Although no direct induction of bone formation by C91 has been reported, it can rescue iPSC cells from Goucher disease patients with impaired osteogenic differentiation due to GBA1 gene mutation and restore the mineralization ability of these cells after osteogenic differentiation (15), due to improved canonical Wnt signaling in these mutant iPSC cells.

Specific inhibition of GSK-3β by small molecules is key to the screening of this class of small molecules. C91 is the most effective and specific inhibitor of GSK-3. It reduced the intensity of CDK2-cyclin A inhibition by nearly 50 times and did not inhibit any other protein kinase at 1 µM (37). Osteoblasts play the most direct and important role in bone regeneration. Bone formation is a relatively complex biological process, which is mainly characterized by the differentiation of bone marrow stem cells into osteoblasts, followed by osteoblast maturation, matrix formation and mineralization. GSK-3β in the cytoplasm phosphorylates β-catenin protein and leads to its ubiquitination, which is finally brought to polysome degradation, thus preventing the transmission of canonical Wnt signaling, so that the cells cannot complete osteoblastic differentiation. GSK-3 inhibitors, on the other hand, inactivate GSK-3B, thereby stabilizing β-catenin in the cytoplasm, allowing β-catenin protein to enter the nucleus and activate canonical Wnt signaling target genes. Therefore, as a highly specific and functional GSK-3 inhibitor, activation of Wnt signaling by C91 can be used to promote the osteogenic differentiation of cells and carry out translational studies.

MDC staining using markers LC-3I/II and autophagosomes demonstrated that C91 induces autophagy during osteoblast differentiation and mineralization. In this study, we innovatively found that canonical Wnt signaling activates autophagy and is involved in the regulation of osteoblast differentiation. Autophagy can degrade damaged organelles and foreign bodies to form phosphate, which is secreted into the cytoplasm, providing components for biomineralization (38). Autophagy has been implicated in the differentiation of osteoblasts. It has been demonstrated that autophagy-related proteins are involved in osteoblast differentiation and bone formation, and other studies have reported that autophagy affects osteoblast mineralization and osteocyte network structure (39, 40). Mouse models knocking out autophagy genes have shown a significant reduction in osteophagy volume, number, and thickness (41, 42). There is increasing evidence for the role of autophagy in the development and progression of osteoporosis, and studies of genome-wide association data in humans have reported that autophagy-related genes are associated with osteoporosis (16, 17). In addition, the reduction of autophagy can promote osteoporosis by increasing oxidative stress (43, 44). In this study, we found that C91 initiates autophagy in ST2 cells by activating canonical Wnt signaling, and autophagy promotes osteogenic differentiation by increasing β-catenin levels. So autophagy did not rescue the inhibition of C91-induced osteoblast differentiation by Triptonide, an inhibitor of Wnt target gene transcription. The finding can be used for the treatment and prevention of osteoporosis.

Although C91 activates canonical Wnt signaling and is able to determine cell fate, it is not an oncogene for bone organs. Among GSK-3 inhibitors, C91 is less cytotoxic. Because the Wnt/β-catenin signaling pathway is essential for many biological responses, targeting the safety of this pathway is the primary precondition for development for therapeutic purposes (45). For Wnt signaling pathway activators, there is concern whether it induces tumors because activating Wnt/β-catenin signaling alters cell fate after all. Notably, tumors triggered by Wnt signaling have only been detected in the intestine in humans and mice with APC mutations that are aberrantly activated by Wnt/β-catenin signaling, and tumors detected in other organs or tissues are almost unreported (46, 47). The fact suggests that local use of Wnt activators in organs or tissues other than the intestine is a safe treatment (48). No tumors have been reported in the treatment of neurological diseases with LiCl for more than sixty years.It was found in a potential study of four commonly used GSK-3 inhibitors on the activation of the canonical Wnt signaling pathway by mouse embryonic stem cells.C91 and SB-216763 have low toxicity in mouse embryonic stem cells, while BIO has high toxicity (49).The IC50 of BIO was 0.48 µM, that of SB-216763 was 5.7 µM, and that of C91 was 4.9 µM. Naujok et al. suggested that C91 is the small molecule that activates the Wnt signaling pathway to the greatest extent, but is least cytotoxic (50). C91 had no significant effect on the normal growth and proliferation of ST2 cells at 5 µM, and the survival rate of 7 days culture in three dimensions was as high as 96.4%, demonstrating that the small molecule C91 has low cytotoxicity.

C91 at concentrations of 5 μM and below had no effect on the proliferative activity of ST2 cells, with only 7.5 and 10 μM of C91 elevating cell proliferative viability (**Figure 1A**). C91 was released on 3D printed GelMA hydrogels, 27% of which was released on day 1, and the C91 concentration reached 9.4 μM. Although the release rate decreased by about 20 ~ 40% per day (data not shown) later, high concentration of C91 increased the proliferative activity of ST2 cells. Culture 7 days released 83% of the total amount, so linear proliferation was produced from day 1 to day 7 compared with the vehicle DMSO control, but released C91 resulted in a continuous increase in cell proliferation activity (**Figure 7E**). Therefore, a decrease in C91 concentration without additionally increasing the proliferative activity of the cells could be considered to reduce the possibility of tumors. Reducing the concentration of C91 also did not affect the survival of the cells, as the cell survival rate of the vehicle DMSO control was also as high as 96.4% (**Figures 7B, C**). So the high survival rate of cells solves the industrial problem of cell survival and growth in hard materials with three-dimensional structure. This strongly ensures the osteogenic differentiation of subsequent cells, and on the 7th day, osteogenic differentiation medium was used instead to allow osteogenic differentiation and mineralization of proliferating ST2 cells (**Figure 8D**). Therefore, this way of use is safe and effective and can be translated and applied to the repair of bone defects.

The small molecule C91 promotes osteogenic differentiation and mineralization by activating the Wnt/β-catenin signaling pathway in ST2 cells. At the same time, C91 induces autophagy and activates the Wnt/β-catenin pathway to promote osteogenic differentiation. The 3D biological culture simulates the osteogenesis process *in vivo*, and the sustained release of C91 on the 3D module verifies its osteogenic differentiation and biomineralization functions. The small molecule C91 is a safe and effective pro-osteogenic small molecule, which may provide more options for the clinical treatment of osteoporosis and the local release of C91 for the treatment of bone defects in the future.

## Data Availability Statement

The original contributions presented in the study are included in the article/supplementary material. Further inquiries can be directed to the corresponding author.

## Author Contributions

Conceptualization, BW and XT. Methodology, BW, SK, and PW. Investigation, BW, SK, XW, and PW. Data analysis, BW, SK, JC, YL, and XT. Writing—original draft preparation, BW, YL, and JC. Writing—review and editing, BW, YL, and XT. Funding acquisition, XT. Resources, XT. Supervision, XT. All authors contributed to the article and approved the submitted version.

## Funding

This research was funded by the National Natural Science Foundation of China U1601220, 81672118, and 82072450 (XT), and Chongqing Science and Technology Commission—Basic Science and Frontier Technology Key Project cstc2015jcyjBX0119 (XT). The Chongqing Postgraduate Research and Innovation Project (CYB20167, CYS20219).

## Conflict of Interest

The authors declare that the research was conducted in the absence of any commercial or financial relationships that could be construed as a potential conflict of interest.

## Publisher’s Note

All claims expressed in this article are solely those of the authors and do not necessarily represent those of their affiliated organizations, or those of the publisher, the editors and the reviewers. Any product that may be evaluated in this article, or claim that may be made by its manufacturer, is not guaranteed or endorsed by the publisher.
